# The Community In-Reach and Care Transition (CIRACT) clinical and cost-effectiveness study: study protocol for a randomised controlled trial

**DOI:** 10.1186/s13063-015-0551-2

**Published:** 2015-02-08

**Authors:** Alison Watson, Lisa Charlesworth, Ruth Jacob, Denise Kendrick, Philippa Logan, Fiona Marshall, Alan Montgomery, Tracey Sach, Wei Tan, Maria Walker, Justin Waring, Diane Whitham, Opinder Sahota

**Affiliations:** Queens Medical Centre, Nottingham University Hospitals, Derby Road, Nottingham, NG7 2UH England; Nottingham Clinical Trials Unit|, University of Nottingham, Queens Medical Centre, Derby Road, Nottingham, NG7 2UH England; Health Sciences, University of Warwick, University Road, Coventry, CV4 7AL England; School of Medicine, Faculty of Medicine and Health Sciences, University of Nottingham, Queens Medical Centre, Derby Road, Nottingham, NG7 2UH England; Nottingham University Business School, Jubilee Campus, Nottingham, NG8 1BB England; School of Medicine, Health Policy and Practice, Norwich Medical School, University of East Anglia, Norwich Research Park, Norwich, NR4 7TJ England; Nottingham City Care Partnership, 1 Standard Court, Park Row, Nottingham, NG1 6GN England

**Keywords:** Rehabilitation, In-reach, Community, In-patients, Older people

## Abstract

**Background:**

Older people represent a significant proportion of patients admitted to hospital. Their care compared to younger patients is more challenging, length of stay is longer, risk of hospital-acquired problems higher and the risk of being re-admitted within 28 days greater. This study aims to compare a Community In-Reach and Care Transition (CIRACT) service with Traditional Hospital Based rehabilitation (THB-Rehab) provided to the older person. The CIRACT service differs from the THB-rehab service in that they are able to provide more intensive hospital rehabilitation, visiting patients daily, and are able to continue with the patient’s rehabilitation following discharge allowing a seamless, integrated discharge working alongside community providers. A pilot comparing the two services showed that the CIRACT service demonstrated reduced length of stay and reduced re-admission rates when analysed over a four-month period.

**Methods/Design:**

This trial will evaluate the clinical and cost-effectiveness of the CIRACT service, conducted as a randomised controlled trial (RCT) with an integral qualitative mechanism and action study designed to provide the explanatory and theoretical components on how the CIRACT service compares to current practice. The RCT element consists of 240 patients over 70 years of age, being randomised to either the THB therapy group or the CIRACT service following an unplanned hospital admission. The primary outcome will be hospital length of stay from admission to discharge from the general medical elderly care ward. Additional outcome measures including the Barthel Index, Charlson Co-morbidity Scale, EuroQoL-5D and the modified Client Service Receipt Inventory will be assessed at the time of recruitment and repeated at 91 days post-discharge. The qualitative mechanism and action study will involve a systematic programme of organisational profiling, observations of work processes, interviews with key informants and care providers and tracking of participants. In addition, a within-trial economic evaluation will be undertaken comparing the CIRACT and THB-rehab services to determine cost-effectiveness.

**Discussion:**

The outcome of the study will inform clinical decision-making, with respect to allocation of resources linked to hospital discharge planning and re-admissions, in a resource intensive and growing group of patients.

**Trial registration:**

Registered with the ISRCTN registry (ISCRCTN94393315) on 25 April 2013 (version 3.1, 11 September 2014).

**Electronic supplementary material:**

The online version of this article (doi:10.1186/s13063-015-0551-2) contains supplementary material, which is available to authorized users.

## Background

The number of people aged 70 years and over in the United Kingdom is expected to double by 2025 [1], with an associated increase in the proportion of unplanned medical admissions in this age group over the next 10 years [2]. Many of these patients have multiple co-morbidities. These include poly-pharmacy, cognitive deficits and physical impairments which makes their care, management and timely discharge from hospital complex. This often prolongs length of stay and increases 28 day re-admission rates in older patients when compared to their younger counterparts [3].

Nationally, over the last six years, length of stay has reduced but 28 day re-admission rates have increased from 11 to 14% [4]. In Nottingham, between 2010 and 2013 the mean length of stay across the medical elderly care wards (five wards, 6,924 patients) decreased from 14 to nine days, but 28 day re-admission rates increased from 14 to 19% (Sahota and Fleming, 2013, unpublished data). The reasons for these readmissions are multi-factorial. Patients are often unprepared for their self-management role in the next care setting [5] and are often unable to reach an appropriate healthcare practitioner who has access to their discharge summary when problems arise [6-8]. There simply may not be appropriate resources in the community to respond to the needs of these patients in a responsive manner, placing them at risk of readmissions. Furthermore, patient safety is often compromised during this vulnerable period, with high rates of medication errors [9-12], incomplete or inaccurate information on transfer [13] and lack of appropriate follow-up care [14], collectively leading to fragmented discharge planning and increased rates of recidivism to high intensity care settings when patients’ care needs are not met [15]. This increases health and social care costs, which will be further exacerbated by government plans to reduce funding if patients are re-admitted as an emergency within 28 days of being discharged [16].

In England and Wales, to address the problem of rising re-admission rates, the Department of Health has allocated £300 million in 2014, as part of the ‘funding for re-ablement linked to the hospital discharge’ funding stream [17]. This money is to be spent on developing local plans in conjunction with the Local Authority, Foundation Trusts and NHS Trusts and Community Health services, to facilitate seamless care for patients on discharge from hospital and prevent avoidable hospital readmissions. Some Clinical Commissioning Groups (CCGs) have invested in ‘early supported discharge at home’ schemes, some into ‘community-based rehabilitation’ schemes and some have invested very little at all. Reviews of the literature suggest that it is currently unclear which is the most effective and efficient structure and organisation of community and intermediate care services in relation to their purpose [18,19].

We undertook a structured literature review to identify systematic and Cochrane reviews of ‘early supported discharge’, ‘discharge planning from hospital to home’ and ‘care transition’ interventions [5,10,18-23]. The key components in the successful RCTs of high methodological quality were then identified as more intensive rehabilitation, working closer with the patient and their relatives and facilitating ‘care transition’ back into community, informing the frequency and duration of the service intervention.

This was followed by a series of multi-perspective focus group meetings with service users, experienced healthcare professionals and service managers. The key aims and objectives of these meetings were to identify the make-up of the team and the delivery of the intervention. Important areas identified the need for generic rehabilitation staff, a dedicated allied healthcare ‘transition coach’ and senior leadership and governance within the team.

The ‘care transition model’ has been proposed as a model to address potential threats to patient quality and safety during care transition [22], and has been shown to reduce early hospital re-admission rates [5]. Care transition is defined as a set of actions ensuring the coordination and continuity of care as patients transfer between different locations. These include logistical arrangements, education of the patient and family and coordination among the health professionals involved in the transition essential for persons with complex care needs. Key to this model is a home visit, ideally within 48 to 72 hours after hospital discharge. The aim of the visit is to ensure that the patient has received appropriate services and equipment, coordinate communication between primary and secondary care and review the patient’s goals established on discharge from hospital.

Provision for elderly medical inpatients in Nottingham takes two forms. One consists of the Traditional Hospital Based multidisciplinary rehabilitation (THB-rehab) service, referring the patient on to different community rehabilitation and social services on discharge. The other is a pilot Community In-Reach rehabilitation and Care Transition (CIRACT) service, consisting of a senior occupational therapist, senior physiotherapist, social services practitioner and an assistant practitioner, working more closely across multiple boundaries with patients and their carers. The CIRACT intervention is set apart from other models of care as although the team is based on the hospital ward, they are employed by a community NHS provider (NHS Nottingham City Care Partnership), and therefore are able to bring their community expertise into the hospital care setting, bridging the gap between the community and hospital settings. In addition, the CIRACT service aims to i) provide intensive rehabilitation; ii) work closely with the patient, relatives and community-based health and social care professionals to facilitate and access a wider range of care and services in the community and iii) provide a community follow-up service.

Pilot data suggest that compared to the THB-rehab service, the CIRACT service can reduce median length of stay and 28 day re-admission rates, over a four-month period. However the clinical and cost-effectiveness of the service needs to be evaluated in a randomised trial.

### Objectives

The primary objective of the CIRACT trial is to assess whether length of hospital stay for unplanned hospital admission among people aged 70 years or older allocated to receive the CIRACT service is different compared with the THB-rehab. The secondary objectives include assessing the effects of the CIRACT service on re-admission rates within 28 days post-discharge; super spell bed days (total time in NHS care); patient function and patient health-related quality of life. Alongside this, the qualitative part of the trial aims to investigate the design and delivery of the CIRACT service and assess service user experiences of the two forms of rehabilitation delivery. The health economics part of the study aims to evaluate the cost-effectiveness of the CIRACT service compared to the THB-rehab service.

### Trial design

The study is a single-centre pragmatic parallel randomised controlled trial with an integral qualitative mechanism and action study and health economic study. Participants will be followed-up to 91 days post-discharge.

## Methods/Design

The initial part of the study including recruitment, treatment allocation and in-patient rehabilitation will take place on the acute hospital ward. Follow-up data will be collected via a telephone interview with the participant or personal consultee in their own home.

### Study setting

Any patient admitted to the hospital from the medical admissions unit to one of the three acute, general medical elderly care wards at the Queen’s Medical Centre (1,800 beds serving a population of 680,000), Nottingham, United Kingdom will be screened for inclusion into the trial.

### Eligibility criteria

Participants will be eligible for the study if all of the inclusion and none of the exclusion criteria are met.

#### Inclusion criteria

The inclusion criteria are as follows:

1. aged 70 years and over,
2. have been admitted to hospital on the general medical elderly care ward as an unplanned admission,
3. have been admitted to hospital from their own home or residential care home and
4. have their registered GP within the Nottingham City CCG catchment area.

#### Exclusion criteria

The exclusion criteria are as follows:

1. were bed bound prior to admission or moribund on admission,
2. are receiving palliative care,
3. were previously included in the trial on an earlier admission or
4. are not able to be screened and recruited by the Research Assistant (RA) within 36 hours of admission to the study ward. A 36-hour deadline ensures there is not a delay to the participant receiving therapy, and will enable the recruitment of a large proportion of patients admitted over a weekend, when no RA will be available recruit.

### Study intervention

Each participant will be randomly allocated to the CIRACT service (intervention arm) or THB-rehab (control arm). The CIRACT team will conduct an assessment of the participant’s ability to perform certain tasks within 24 hours of randomization, enabling the formulation of a comprehensive rehabilitation plan. The CIRACT team will consist of a physiotherapist, an occupational therapist and a community care officer who will be employed by Nottingham CityCare, but will be based on one of the study wards. While in hospital the participants will be treated daily (seven days a week, if appropriate) by the CIRACT team and the time of rehabilitation they receive will be dependent on their needs. During the participant’s hospital stay the CIRACT team will liaise with the participant and their carer(s) to visit the participant’s home to assess and provide recommendations for equipment and make adaptations and/or modifications if required. The CIRACT service will utilise the teams’ expertise in community working to form links with the appropriate services to ensure a smooth and effective discharge. In more complex cases the CIRACT team will take the participant out of the hospital for a home visit prior to discharge. Following discharge, the CIRACT team will visit the participant at home within 48 hours of discharge to assess the level of rehabilitation required at home, and the CIRACT team will undertake further follow-up visits as deemed necessary.

The THB-rehab service will be provided by the hospital-based therapy teams on weekdays only as a control group receiving ‘usual care’. Members of these teams will jointly conduct an assessment of the participant’s ability to perform certain tasks and provide recommendation for rehabilitation. The ward team will refer the participant to the appropriate community-based services for the provision of equipment at home, personal care and ongoing rehabilitation where appropriate at discharge. Once discharged from hospital, the patient will have no contact with the ward rehabilitation staff.

In either group, if a participant becomes medically unwell at any point to the extent they are no longer able to undertake therapy (as directed by the ward doctor), the treating team will withhold further therapy until being instructed by the ward doctor it is safe to recommence therapy. The nursing and medical care provided by the ward staff will not differ between the two groups.

### Primary outcome measures

The primary outcome is hospital length of stay from randomisation to discharge from a general medical elderly care ward. The date of randomisation will be used instead of date of admission to the ward to negate for the impact of a delay in randomisation and reduce the risk of cross contamination.

### Secondary outcome measures

The secondary outcome measures are:

1. Super spell bed days (total time in NHS care including hospital care and intermediate care) from admission to 91-day follow-up.
2. Unplanned re-admission rates at day 28 and day 91. Days 28 and 91 were selected as they are the commissioners’ targets for re-admission.
3. The Barthel ADL index [24] at baseline and at 91 days post-discharge to assess activities of daily living (ADL) and mobility.
4. EQ-5D-3 L health-related quality of life measure [25] at baseline and at 91 days post-discharge. The EQ-5D-3 L is a standardized measure of quality of life looking at mobility, self-care, usual activities, pain and/or discomfort and anxiety and/or depression.
5. The Charlson comorbidity index [26] at baseline and at 91 days post-discharge to assess comorbidity. The Charlson codes a total of 22 comorbid conditions into a single score.
6. The mean cost per patient of CIRACT and THB-rehab will be estimated using micro-costing methods.
7. The modified Client Service Receipt Inventory (CSRI) questionnaire [27] at baseline and at 91 days post-discharge. The CSRI is a cost questionnaire designed to measure and compare the service cost of both rehabilitation services.

**Figure 1:**
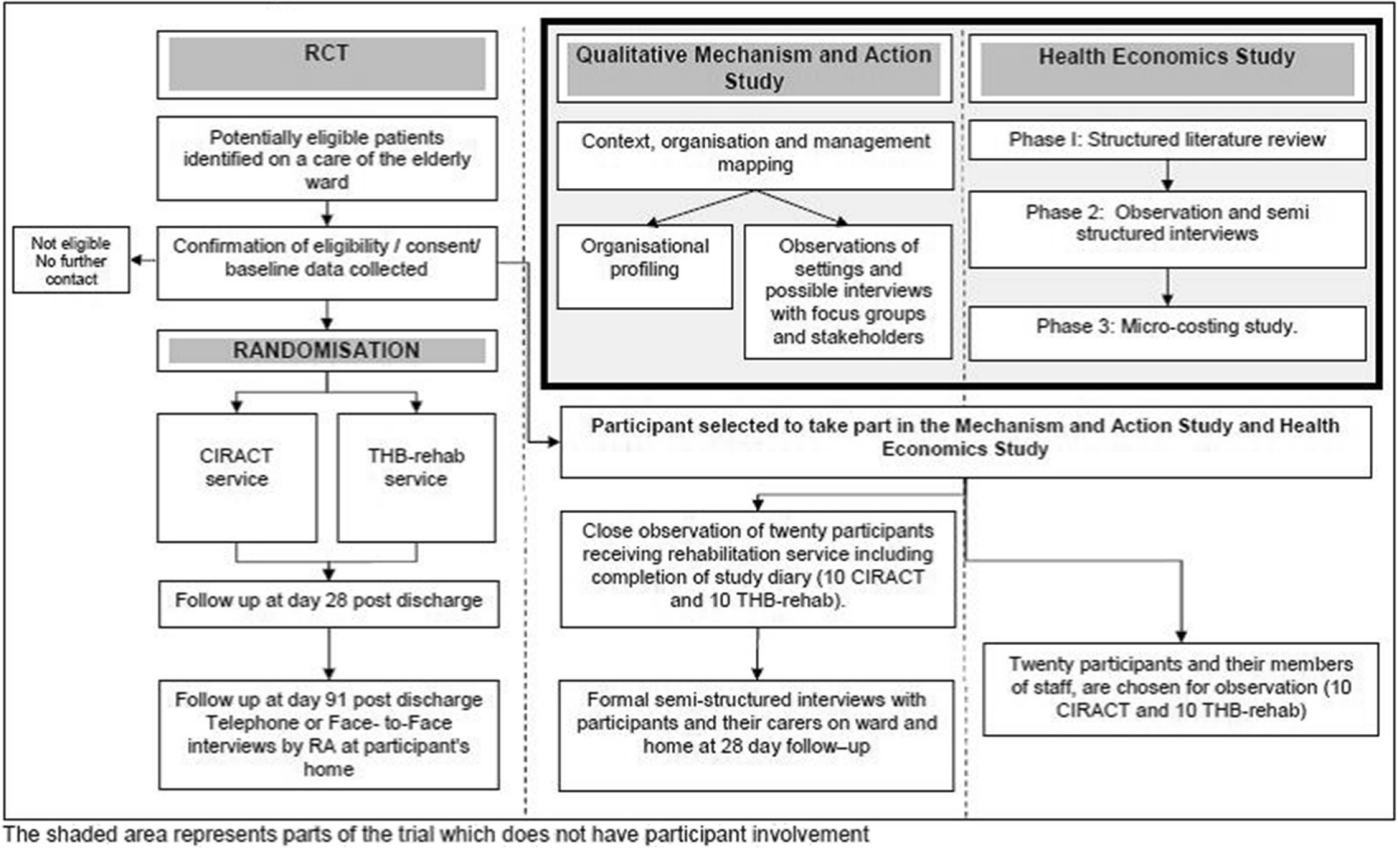
**Trial flow diagram.** Shaded areas represent parts of the study which do not have participant involvement. CCI = Charlson Comorbidity Index, CIRACT = Community In-Reach and Care Transition, CSRI = Client Services Record Inventory, EQ-5D-3 L = EuroQol-5 dimension-3 Level quality of life scale, MMSE = Mini Mental State Examination, THB-Rehab = Traditional Hospital Based Rehabilitation.

The RA will collect demographic data (including age, sex, reason for admission and living circumstances) and outcome measures via face-to-face interviews on trial entry, and via a telephone interview at the three-month follow-up assessment. The RA will be blinded to treatment allocation. The time schedule of enrolment, interventions, assessments and visits for participants is shown in Additional file 1 and the trial flow diagram is presented in Figure 1.

### Sample size calculation

The primary statistical analysis will compare length of hospital stay for those allocated to receive the CIRACT service versus the THB-rehab service. Pilot data showed the log transformed length of stay to be normally distributed, with a standard deviation of 0.9. Therefore, 111 patients per arm will be required for analysis in order to detect a clinically important effect size of three days (equivalent to a ratio of geometric means of 0.7) with two-sided alpha of 5% and 80% power. Allowing for 5% non-collection of primary outcome data, 240 patients in total will be randomised following an acute unplanned admission to hospital over a 13-month recruitment period.

### Recruitment and consent

All eligible participants will be made aware of the trial at the time of admission and invited to participate in the trial. Written informed consent will be obtained by the RA in accordance with the International Conference on Harmonisation Guidelines for Good Clinical Practice (ICH GCP) [28]. In patients who are confused (dementia or delirium such that the subject cannot understand the nature of consenting to a research study and the study process), consent will be obtained by carers or other proxies including staff, following the established framework of Berghmans and Ter Meulen [29], used in previous ethically approved studies in older persons with dementia.

### Randomisation procedure

Once the RA has gained consent from the participant, subjects will be allocated to the CIRACT service or the traditional hospital rehabilitation service (control) arm using the web-based randomisation service provided by the Clinical Trials Support Unit, University of Nottingham.

Randomisation will be determined by a computer-generated pseudo-random code using random permuted blocks of randomly varying size, created by the Nottingham Clinical Trials Unit (CTU), in accordance with their standard operating procedure (SOP) and held on a secure server. Participants will be allocated with equal probability to either arm of the study. The randomisations will be requested through a PC with internet explorer and internet access. The system is located on a dedicated secure server within the University of Nottingham. All communications between the user's PC and the server will be fully encrypted (secured SSL 128-bit encrypted) and via a unique username and password.

### Blinding

The RA collecting data and the research team analysing the data will be blinded to treatment allocation. The participant and ward staff will not be blinded to treatment allocation as the treating therapists will be liaising closely with ward staff to ensure optimal patient care. The three-month follow-up data will be collected by an RA blinded to treatment allocation.

### Qualitative, implementation and action study

The qualitative, implementation and action study aims to understand the situated activities and interactions that constitute the CIRACT service in comparison to THB-rehab service, and to understand how these mechanics of care interact with other factors located in the wider care system. This will investigate how the service is perceived by all participants in terms of transitional planning, effective therapy delivery and the general organisational aspects in relation to the concurrent services provided within the research wards.

The qualitative appraisal also draws upon recent developments in the field of implementation science. It is widely recognised that new interventions or ways of working do not easily move from policy into practice, but are transformed and even inhibited by the contexts into which change is desired, especially professional boundaries [30]. Recent developments in implementation science have led to new approaches for understanding this process, such as the Promoting Action on Research Implementation in Health Services (PARIHS) framework and Normalisation Process Theory. This appraisal of CIRACT was informed by Damschroder *et al*.’s [31] consolidated framework. This draws attention to a range of key factors that frame and are involved in the implementation of new practices including:

1. Characteristics of the intervention itself.
2. The outer and inner context (outer context referring to the wider institutional, political and social environment, with the inner context referring to the internal organisational environment where change is to be made).
3. The role of individuals and groups within the given context including their sense-making, mind-sets, cultures and networks.

A range of behavioural theories inform analysis of individual behaviours, but this study adopts a socio-cultural practice perspective that considers how social practices are embedded within wider social and cultural fields of influence, whilst allowing for individual agency. This is especially relevant in healthcare settings given the influence of professional cultures and boundaries on practices. The final aspect is the implementation processes itself which cuts through involves the above factors. The model also suggests that implementation is non-linear and can cut across organisational and practice levels.

#### Nested qualitative appraisal

The aim of the qualitative, implementation and action study is to understand how the two forms of rehabilitation service (CIRACT service and THB-rehab service) are delivered as a complex intervention in older patients.

In considering the implementation, organisation and delivery of CIRACT, this model draws analytical attention to: i) the nature of CIRACT including how it is configured, disseminated and translated into practice settings; ii) the role of wider changes within the organisation and delivery acute and community care, including wider political issues, financial and regulatory issues; iii) the influence of local organising factors such as resources and staffing, modes of working and professional boundaries and iv) the role of local actors, especially CIRACT and ward staff, but also patients and carers.

This will involve three linked activities (A to C), linked to three research techniques (D to F).

A. Each rehabilitation service will be described and analysed to understand the local context factors that might shape service delivery, including staffing and resource levels, participant group type and variability, provision of support services, service culture, receptivity to change and team/and professional work dynamics.
B. In a purposive selection of participants, the organisation and delivery of the intervention will be described and analysed in-depth to understand how the service is configured and how therapy is planned and delivered.
C. The views and experiences of professionals and participants at each participating site will be described and analysed to understand their involvement in, reactions to and continued use of both forms of rehabilitation.
D. Organisational profiling: involving a relatively standardised checklist pro forum of known contextual and organisational variables known to influence implementation of change and the delivery of complex intervention. This will be completed through focussed visits, guided tours and key stakeholder interviews. These will be undertaken over a two to three day period and recorded using a standard form to facilitate rapid data analysis and comparison.
E. More detailed observations of service organisation and delivery over a period of three to four weeks within a purposive selection of participants. This will be informed by the principles of ethnographic observation, with the research undertaking prolonged periods of direct observation of how the service is organised and delivered.
F. Qualitative interviews and focus groups with representative groups of professionals and participants to understand their shared and distinct experiences of service delivery of each form of rehabilitation.

All interview and focus group data will be analysed following the conventions of framework analysis [32-34]. This is a hierarchical, matrix-based method developed for applied or policy relevant qualitative research. It is a highly structured, transparent and rigorous approach to qualitative data which is well suited to research where timescales are limited, and the goals of research are clearly defined at the outset.

### Health economic study

The health economic study will undertake a within-trial cost-effectiveness and cost utility analysis using established methods [35]. The CIRACT service will be compared to the THB-rehab service from a NHS and Personal and Social Services (PSS) perspective in order to help inform commissioning decisions.

In addition to measuring the cost of the intervention, the resources consumed in the two arms of the study are likely to differ, as there is the potential for differential equipment requests and differential lengths of stay in hospital. Resources will be valued using published unit cost data or where needed using locally derived unit costs.

### Cost analysis

A detailed cost analysis of the two services to the NHS will be considered from a time and motion study, and compared against cost estimates based on standard methods of collecting resource use information (here via a modified Client Service Receipt Inventory (CSRI) questionnaire [27] at baseline and at 91 days post-discharge). The detailed micro-costing study will be conducted in three phases:

1. A structured literature review (phase one): The review will provide information on how to conduct the time and motion study (phase three).
2. Observation and semi-structured interviews (phase two): Observations and interviews will provide information on how to conduct the time and motion study (phase three) and define what activities are undertaken by each service. The observations of the therapists will enable a greater understanding of what each team offers and how they differ in their service provision. This will allow comparisons to be drawn between the two groups.
3. Micro-costing study (phase 3): using the information from phase one and two a time and motion study will be conducted with 10 patients in each arm, recruited through purposive sampling, over a six-month period. Within each treatment arm, we will aim to include half of the participants with baseline admission Barthel ADL of less than 14, and the other half with a Barthel ADL of greater than 14, in order to ensure a wide range of abilities are observed. The time and motion study will record therapy interactions with the patient (face-to-face and indirect) to enable costing of each service provision. To get this data an RA will contact the ward therapists or the CIRACT team (depending on allocation) on a twice daily basis and ask how much time they have spent in direct face-to-face contact with the patient and indirectly, through non-face-to-face contact (ward rounds, telephone calls, referrals, and so forth).

To capture the contacts with therapists post-discharge, the RA will establish from the therapists if they have been referred on for further therapy and, if so, the contact details of the community-based team. The RA will then contact the treating therapist in the community to gather contact time (non-face-to-face may then include travel time) for two weeks following discharge. This will provide us with the data required to cost the contact time for each patient and enable comparisons between groups. The specific activities undertaken by the therapists with the patient will be explored through phase two.

#### Outcome measurement

We will use the EQ-5D-3 L and convert scores into quality-adjusted life years (QALYs) using linear interpolation and area under the curve methods for the trial period [36]. We will adjust for any baseline differences between treatment groups [37]. The main outcome for the cost utility analysis will therefore be incremental cost per QALYs estimated from the change in EQ-5D-3 L score at baseline and at 91 days follow-up, post-discharge.

#### Economic analysis

The results of the analysis will be presented using the incremental cost effectiveness ratio (ICER), if appropriate, with decision uncertainty represented via cost effectiveness acceptability curves (CEACs) based on nonparametric bootstrapping of cost and effect pairs [38]. This will provide robust results to inform decision-makers about whether the NHS should provide the CIRACT service or not compared to traditional care.

### Data collection

Data will be collected using CIRACT trial data collection forms which will be monitored by the Nottingham Clinical Trials Unit (NCTU) for consistency, validity and quality. Missing data and data queries will be referred promptly back to the recruiting site for clarification. If a participant withdraws from the study, the data collected up to that point will be used (as specified in the consent form) but no further data will be collected about that participant. Participants will not be replaced if they withdraw from the study. All reasonable attempts will be made to contact any participant lost to follow-up during the course of the trial in order to complete assessments.

### Data management

All trial data will be entered on a trial-specific database with participants identified only by the unique trial number, date of birth and initials. The database will be developed and maintained by the trial coordinating centre at the NCTU. Access to the database will be restricted and secure. Data quality and compliance with the protocol will be assessed throughout the trial by verification of trial data against clinical records, and by data checking for accuracy and internal consistency. For the follow-up phase, identifiable information about participants will be held in a separate database to the trial anonymised data. Access to this information will be restricted to those involved in the follow-up phase, as authorised by the Chief Investigator.

### Statistical analyses

The analysis and reporting of the trial will be in accordance with the Consolidated Standards of Reporting (CONSORT) guidelines. A full statistical analysis plan will developed before any data are analysed. Appropriate descriptive statistics will be used to compare the characteristics of the randomised participants across the trial groups. The primary approach to all comparative analyses will be based on treatment allocated without imputation of missing data. Between-group differences will be estimated using regression models appropriate for outcome type, adjusting for baseline value of the outcome if collected, and paying attention to 95% confidence intervals as well as *P* values. Analysis of subgroups will be agreed with the Trial Steering Committee and all those undertaken will be reported.

### Data monitoring

The day-to-day management of the trial is the responsibility of the Trial Manager, including research staff training and management. Regular monitoring will be performed by the trial Manager according to the Medical Research Council (MRC) Guidelines for Good Clinical Practice (GCP) in Clinical Trials (22). Following written standard operating procedures, the Trial Manager or where required, a nominated designee of the Sponsor, will verify that the clinical study is conducted and data are generated, documented and reported in compliance with the protocol GCP guidelines and the applicable regulatory requirements.

The study finance, documentation (such as assessment forms and operating procedures), data recording and storage is managed by the Trial Manager, who also monitors progress and provides appropriate reports to the Trial Steering Committee. The Principal Investigator will instigate audits of procedures as required and takes overall responsibility for any protocol changes throughout the study.

As the trial is regarded as low risk, the Trial Steering Committee will also act as the trial management group, and the data monitoring committee. The group will be chaired by the Principal Investigator, and will be comprised of investigators, research staff and patient representatives and will monitors all aspects of the conduct and progress of the trial, ensures that the protocol is adhered to, and will take appropriate action to safeguard participants and the quality of the trial.

In the case of an adverse event, the Trial Manager will receive adverse event forms electronically and the group will meet as required in the event of an excessive serious adverse event rate, or if other information is reported to it which raises concern over the safe conduct of the trial. There is no plan for any interim analyses.

### Harms

Data shall be collected for each individual participant with regards to any fall that occurs whilst an inpatient on the ward until time of discharge, and will be classed as an ‘adverse event’. If the fall results in a fracture, confirmed by radiological findings, it will be classed as a ‘serious adverse event’. This was agreed by the Trial Steering Committee. All serious adverse events will be reported to the sponsor within 24 hours of knowledge of the event using the adapted Nottingham University Hospitals NHS Trust reporting forms made specific to this study. The adverse event risks of taking part in the trial is minimal, as the CIRACT service is not a different type of rehabilitation, but a change in delivery of the THB-rehab service the patients would receive as part of their usual care. The CIRACT service has been assessed in a pilot study and no adverse events occurred.

### Ethics

This study has been given favourable opinion by NRES Committee West Midlands- Staffordshire Research Ethics Committee (REC Reference Number: 13/WM/0050) on 27 February 2013. The trial will be conducted in accordance with ethical principles that have their origin in the Declaration of Helsinki 1996, principles of good clinical practice and the Department of Health Research Governance Framework for Health and Social Care. Any protocol amendments will be submitted to the trial sponsor and ethics committee. A register of protocol amendments will be made available in the study protocol.

### Confidentiality

Confidentiality of all participant information will be maintained throughout the trial. Each participant will be assigned a unique trial identification number (based on their initials and recruitment number), allocated at randomisation to be used for data collection forms, trial documents and the trial database. Data collection forms will be treated as confidential documents, and held securely. The Chief Investigator is the custodian of the data. Participants will not be identified in any future publication.

## Discussion

This article describes the protocol of a randomised trial comparing the clinical and cost-effectiveness of the THB-rehab service, with that of the CIRACT service. The outcome of the study will influence clinical decision-making, with respect to allocation of resources linked to hospital discharge planning and re-admissions, in a very resource intensive and growing group of patients.

The theoretical model underpinning the CIRACT service has been based on the Medical Research Council framework, funded by a Research and Development Pump Priming Research award (Nottingham University Hospitals Charitable funds) and re-ablement funding stream (Nottingham City CCG).

Most early and/or supported discharge models in care of the older person involve receiving patients back into the community from hospital, with little or no direct involvement in hospital-based rehabilitation. The novel aspect of the CIRACT intervention compared to other models of care is that although the team will be based on the hospital ward, they will be employed by a community NHS provider enabling the team to bring their community knowledge into the hospital care setting, in addition to providing more intensive rehabilitation, working closer with the patient and their relatives and being able to facilitate and access a wider range of care and services in the community. This is in contrast to the hospital ward-based therapists, employed by the hospital, who may not have the knowledge and direct and/or rapid accessibility of care and services in the community.

From a financial and cost point of view, we know that the NHS is facing unprecedented financial challenges over the next few years. Therefore any new service requires both clinical and cost-effectiveness evidence to support decision-making. However, many research studies lack good quality costing or cost-effectiveness data, and where studies have been published, average cost of events are often assigned using national and centre-specific price weights, which may not reflect true costs. Micro-costing studies involve direct enumeration and detailed costing of each input consumed [39] although are infrequently undertaken in complex interventional studies. Therefore, in addition to the clinical evaluation of the CIRACT service, we propose to undertake a cost and cost-effectiveness evaluation of this service delivery.

One of the main limitations of this study is that the ward staff and participants cannot be blinded to the rehabilitation group allocation as both services are provided within each ward. However, early pilot work [40] demonstrated that participants and staff very quickly become unaware of group allocation, presumably related to the fluid nature of the ward environment and that much of the rehabilitation is undertaken away from the bedside. Baseline data will be collected prior to randomisation and outcome measures collected by a blinded RA to minimize the risk of detection bias. A statistician blind to group assignment will perform the data analysis.

### Patient and public involvement (PPI)

Members of the PPI advisory group will be present at Trial Management Group (TMG) meetings and a service user representative will be part of the Trial Steering Committee. This patient and public involvement will aim to provide guidance and advice from the patient perspective.

### Dissemination policy

The trial will be reported according to the CONSORT guidelines and will contribute to knowledge on the delivery of change across organisational systems, linkages between different care agencies and sectors and detailed resource use and costing associated with complex interventions. Findings will be published in relevant periodicals and updates sent to relevant websites such as the Health Service Journal and the NHS Primary Care Commissioning website. We will also undertake high-impact conference presentations. Participants who requested a copy of the report will be sent a lay summary of the study. A publication policy will be agreed with co-applicants and a systematic plan, including authorship, for the peer-reviewed publications.

### Trial status

Recruitment to CIRACT commenced in June 2013 and is ongoing at the time of manuscript submission. The expected time of recruitment completion is August 2014.

### Major protocol amendments

Over the course of the trial a number of protocol amendments were made as summarised in Table 1.

**Table 1:** **Summary of major protocol amendments**

**Date of amendment**	**Wording pre-amendment**	**Wording post-amendment**
14 October, 2013	Follow-up at day 91 post-discharge face-to-face interviews by RA at participant’s home.	Follow-up at day 91 post-discharge.
		Telephone or face-to-face interviews by RA at participant’s home.
14 October, 2013	Follow-up to be completed at day 91 (+/− 3 days) post-discharge.	Follow-up to be completed at day 91 (−7/+28 days) post- discharge.
14 October, 2013	Patients excluded at discharge will be excluded from the length of stay (LOS) analysis, but a sensitivity analysis using proxy LOS models will be used to check the robustness of the exclusion.	The final intention-to-treat analysis will include all randomised participants for whom the follow-up assessment of the primary outcome measure is available. Per protocol analysis will include all randomised participants who are deemed to have no protocol violations.
14 October, 2013	Additional paragraph.	Modified client services receipt inventory: GP practices may be contacted by the research team to confirm visit data supplied by the participant.
10 March, 2014.	We propose to conduct the trial on two wards which will give the required numbers within our proposed timetable for the study.	We propose to conduct the trial on two to three wards which will give the required numbers within our proposed timetable for the study.
10 March, 2014.	Additional paragraph.	The RA will contact the therapist involved with looking after the patient at two time points during the day, from admission onto the ward until the patient is discharged from the ward, and continuing if applicable into the participant’s place of residence for a period of two weeks Data collected will be coded and entered into the database recording total time spent with the patient (face-to-face and non-contact). A summary of location codes will be extrapolated from phase two.

## Additional file

Additional file 1:
**Schedule of enrolment, interventions and assessments in the CIRACT trial.**

## Abbreviations

ADL: Activities of daily living
CCG: Clinical Commissioning Group
CIRACT: Community In-Reach and Care Transition
CSRI: Client Service Receipt Inventory
EQ-5D: EuroQol group’s five dimensions of quality of life measure
NCTU: Nottingham Clinical Trials Unit
NHS: National Health Service
NIHR: National Institute of Health Research
NRES: National Research Ethics Service
PPI: Patient and Public Involvement
PPS: Prospective payment system
PSS: Patient and social services
RA: Research Assistant
RCT: Randomised controlled trial
REC: Research Ethics Committee
THB-rehab: Traditional hospital-based rehabilitation
TMG: Trial Management Group
