# Supplementary material for: The Community In-Reach and Care Transition (CIRACT) clinical and cost-effectiveness study: study protocol for a randomised controlled trial
Source: Trials. 2015 Feb 8;16:41. doi: 10.1186/s13063-015-0551-2 (PMC4327808; doi:10.1186/s13063-015-0551-2)
Supplement: Additional file 1: — Schedule of enrolment, interventions and assessments in the CIRACT trial. [file 13063_2015_551_MOESM1_ESM.doc]

*Table 1: Schedule of enrolment, interventions, and assessments in the CIRACT trial. (MMSE= Mini Mental State Examination, CCI= Charlson Comorbidity Index, EQ-5D-3L= EuroQol-5 dimension-3 Level quality of life scale, CSRI=Client Services Record Inventory, THB-Rehab= Traditional Hospital Based Rehabilitation, CIRACT= Community In-Reach And Care Transition).*

|  | **STUDY PERIOD** | | | |
| --- | --- | --- | --- | --- |
|  | **Enrolment** | **Allocation** | **Post-allocation** | |
| **TIMEPOINT** | ***<36hrs form Admission to ward*** | **Day 0** | ***Discharge*** | ***91 days from discharge*** |
| **ENROLMENT:** |  |  |  |  |
| **Eligibility screen** | X |  |  |  |
| **Informed consent** | X |  |  |  |
| ***MMSE*** | X |  |  |  |
| ***Barthel*** | X |  |  | X |
| ***CCI*** | X |  |  | X |
| ***EQ-5D-3L*** | X |  |  | X |
| **Modified CSRI** | X |  |  | X |
| **Allocation** |  | X |  |  |
| **INTERVENTIONS:** |  |  |  |  |
| ***THB-Rehab (Control)*** |  |  |  |  |
| ***CIRACT Service (intervention*** |  |  |  |  |
| ***FOLLOW UP:*** |  |  |  |  |
| ***Visit/Telephone call*** |  |  |  | X |
